# Synergism of Mild Heat and High-Pressure Pasteurization Against *Listeria monocytogenes* and Natural Microflora in Phosphate-Buffered Saline and Raw Milk

**DOI:** 10.3390/microorganisms6040102

**Published:** 2018-10-03

**Authors:** Abimbola Allison, Shahid Chowdhury, Aliyar Fouladkhah

**Affiliations:** 1 Public Health Microbiology Laboratory, Tennessee State University, Nashville, TN 37209, USA; abimbolaallison20@gmail.com (A.A.); schowdh1@tnstate.edu (S.C.); 2 Cooperative Extension Program, Tennessee State University, Nashville, TN 37209, USA

**Keywords:** *Listeria monocytogenes*, natural background microflora, raw milk, high-pressure pasteurization, synergism of mild heat and pressure

## Abstract

As many as 99% of illnesses caused by *Listeria monocytogenes* are foodborne in nature, leading to 94% hospitalizations, and are responsible for the collective annual deaths of 266 American adults. The current study is a summary of microbiological hurdle validation studies to investigate synergism of mild heat (up to 55 °C) and elevated hydrostatic pressure (up to 380 MPa) for decontamination of *Listeria monocytogenes* and natural background microflora in raw milk and phosphate-buffered saline. At 380 MPa, for treatments of 0 to 12 min, *d*-values of 3.47, 3.15, and 2.94 were observed for inactivation of the pathogen at 4, 25, and 50 °C. Up to 3.73 and >4.26 log CFU/mL reductions (*p <* 0.05) of habituated *Listeria monocytogenes* were achieved using pressure at 380 MPa for 3 and 12 min, respectively. Similarly, background microflora counts were reduced (*p <* 0.05) by 1.3 and >2.4 log CFU/mL after treatments at 380 MPa for 3 and 12 min, respectively. Treatments below three min were less efficacious (*p* ≥ 0.05) against the pathogen and background microflora, in the vast majority of time and pressure combinations. Results of this study could be incorporated as part of a risk-based food safety management system and risk assessment analyses for mitigating the public health burden of listeriosis.

## 1. Introduction

The epidemiological evidence, derived from the Centers for Disease Control and Prevention (CDC) active surveillance data, indicates that every year in the United States 1591 illness episodes occur due to infections with *Listeria monocytogenes* [1]. These illnesses are almost exclusively associated with contaminated food products (i.e., about 99% of cases are foodborne in nature) and lead to hospitalization in about 94% of cases. Among those hospitalized, 15.9% die annually, one of the highest mortality rates associated with any foodborne pathogen [1,2]. CDC’s National Outbreak Reporting System (NORS) also delineated that from 1998 to 2016 there have been at least 66 foodborne outbreaks associated with *Listeria monocytogenes*, leading to 852 illnesses with >72% and >15% hospitalization, and death episodes, respectively [3]. Fluid milk had been associated with recent outbreaks of *Listeria monocytogenes* including a 6-month outbreak in Massachusetts in 2007, a 6-month multistate outbreak in 2014, and an 11-month outbreak in Washington in 2014 [3]. This ubiquitous bacterial pathogen has also created a plethora of consumer insecurity and public health challenges globally, including a 2018 foodborne Listeriosis outbreak in South Africa, which has been categorized as the largest foodborne outbreak in the recorded history of food safety [4]. The elderly, the very young, pregnant women, and immunocompromised are particularly considered as the at-risk population for Listeriosis who comprise approximately 30% of the U.S. population [5]. Raw milk, ready-to-eat products, and dairy products prepared using raw milk are some of the main vehicles for this pathogen in the food chain [3,6].

Consumers of the 21st century have evolving demands and expectations from food products, although consumption of raw milk is a major public health concern, many consumers in developed economies are preferring this product due to their perception for greater healthfulness of raw milk, improved digestion, nutritive value, and preferred organoleptic properties [7]. Although the sale of raw milk had been prohibited in several states, many consumers receive the product through legislative loopholes such as “cow-share” programs [7].

With recent advancements in engineering of high-pressure processing units, this technology is gaining rapid adoption across various sectors of food manufacturing for assuring microbiological safety and extending the shelf-life of various products, providing a clean label, as well as fresh-like organoleptic properties of treated foods [8]. As such, the NACMCF (National Advisory Committee on Microbiological Criteria for Foods) has recently recommended redefinition of pasteurization by including high-pressure processing as a non-thermal pasteurization method [9].

The sales of pressure treated products had been in consistent increase in recent years and are expected to surpass $9 billion annually in the United States [10]. Considering consumers’ demand for minimally processed foods, the plethora of foodborne public health episodes associated with *Listeria monocytogenes,* and the increasing momentum in adoption of high-pressure pasteurization in the private food industry, the current study is a microbiological hurdle validation study to investigate synergism of mild heat and elevated hydrostatic pressure for decontamination of raw milk from the pathogen. The study further calculates inactivation indices in raw milk as well as in buffered environment and provides information on decontamination of natural microflora (spoilage organisms) of raw milk as affected by the treatments.

## 2. Materials and Methods

### 2.1. Listeria monocytogenes Strains, Preparation of Culture, and Inoculation

Four strains of *Listeria monocytogenes* (ATCC^®^ numbers 51772, 51779, BAA-2657, 13932) were used for inoculation of raw milk and phosphate-buffered saline in separate experiments. The bacterial strains were chosen due to their public health significance, representing diverse ribotypes, PFGE patterns, serotypes (1/2a, 1/2c, and 4b), and lineages [6]. For each strain separately, a loopful from frozen glycerol stock was aseptically transferred into 10 mL Tryptic Soy Broth (Difco, Becton Dickinson, Franklin Lakes, NJ, USA) supplemented with 0.6% yeast extract (TSB + YE) to minimize the acid stress of the cells during preparation of overnight suspension [11]. This bacteriological medium has also been previously used in high-pressure processing treatments to minimize the effect of acid stress during culturing of bacterial inoculum [12]. The inoculated TSB + YE was then incubated at 37 °C for 22–24 h. One loopful of this overnight suspension was then streak plated onto the surface of Tryptic Soy Agar (Difco, Becton Dickinson, Franklin Lakes, NJ, USA), and incubated at 37 °C for 24 h. The plates were then kept for up to a month at 4 °C prior to the experiment.

Five days prior to the experiments, each strain was activated by transferring a single colony from the above-mentioned plates stored at 4 °C, into 10 mL TSB + YE. After incubation at 37 °C for 22–24 h, a 100 µL aliquot was then aseptically sub-cultured into another 10 mL of TSB + YE and re-incubated at 37 °C for 22–24 h. Cells of each strain (2 mL per strain) were then harvested using centrifugal force at 6000 revolutions per min (3548 *g*, for 88 mm rotor) for 15 min (Model 5424, Eppendorf North America, Hauppauge, NY, USA; Rotor FA-45-24-11). After removal of the supernatant and for further removal of sloughed cell components, excreted secondary metabolites, and growth media, the cells were then re-suspended in Phosphate-buffered Saline (PBS, VWR International, Radnor, PA, USA), then re-centrifuged using the above-mentioned time, intensity, and instrumentation. Then, after discarding the supernatant, to improve the external validity of the challenge study, each bacterial strain was then individually habituated in sterilized milk and/or PBS. Strains were habituated at 4 °C for 72 h to allow acclimatization of the pathogen to low temperature and intrinsic factors of the food/medium [13,14]. On the day of the experiments, the individually habituated strains were then composited and used as inoculum for microbiological challenge studies in raw milk and PBS, at target population levels of 5 to 6 log CFU/mL in the raw milk experiments and 7 to 8 log CFU/mL in the PBS experiments. Fresh raw milk was purchased through a cow-share program from the outskirt of Nashville, TN, stored aseptically in a refrigerated cooler during transportation and were utilized for the experiment less than 24 h after the purchase. Levels of inoculation and elements of experimental design were selected (data not shown) based on preliminary trials.

### 2.2. Mild Heat and High-Pressure Pasteurization

For experiments involving inoculation of raw milk, hydrostatic pressure (Barocycler Hub440, Pressure Bioscience Inc., South Easton, MA) of 380 Megapascal (MPa), i.e., 55,000 pounds per square inch (PSI) and 310 MPa (45,000 PSI) were applied at 4, 25, and 50 °C. Similarly, for experiments involving inoculation of PBS, pressure levels of 380 MPa (55,000 PSI), 310 MPa (45,000 PSI), and 240 MPa (35,000 PSI) were used at 4 °C and 55 °C for time intervals of 0 (untreated control) to 12 min. The pressure intensity levels, as well as temperatures and time combinations, were selected based on preliminary trials [11] and reported based on English and metric units on the graphs due to the popularity of both systems for stakeholders in various regions. The above-mentioned processing unit has chamber size of 16 mL, surrounded with a stainless water jacket connected to a refrigerated circulating water bath (Model refrigerated 1160s, VWR International, Radnor, PA, USA) for precise control of the temperature during the treatments. For monitoring the temperature, two k-type thermocouples (Omega Engineering Inc., Norwalk, CT, USA) were manually inserted inside the wall of the chamber and secured with thermal paste (Model 5 AS5-3.5G, Arctic Silver, Visalia, CA, USA). These thermocouples were connected to the unit’s software (HUB PBI 2.3.11 Software, Pressure BioScience Inc., South Easton, MA, USA) that, in addition to chamber pressure, recorded the temperature values every three seconds [11]. The coolant of the circulating water bath and pressure transmission fluid was distilled water (total soluble solids less than 30 parts per million). The chamber of the unit was purged prior to each analysis for removal of residual air, to assure treatments were hydrostatic in nature. All treatments were conducted inside no disk PULSE tubes (Pressure BioScience Inc., South Easton, MA, USA), containing 1.5 mL of habituated inoculum in raw milk or PBS. It is noteworthy that each reported treatment time excludes the time for pressure increase (come-up time of 3 s) and the release time (come-down time of 1 s). These values were recorded and monitored using the Barocycler mode of HUB PBI 2.3.11 Software (Pressure BioScience Inc., South Easton, MA, USA).

### 2.3. The pH, Neutralization, and Microbiological Analyses

In order to neutralize the intrinsic factors of the food vehicle prior to microbiological enumeration, each sample was neutralized using 3 mL of D/E neutralizing broth (Difco, Becton Dickinson, Franklin Lakes, NJ, USA) per 1 mL of the sample, thus, the detection limit of this study was 0.35 log CFU/mL. After neutralization, for experiments involving the inoculation of raw milk, pressure-treated and untreated controls were 10-fold serially diluted in Maximum Recovery Diluent (Difco, Becton Dickinson, Franklin Lakes, NJ, USA) to maximize the recovery of injured cells. The neutralized diluents were then spread plate onto the surface of PALCAM base agar (Becton, Dickinson and Company, Sparks, MD, USA) supplemented with Ceftazidime (Becton, Dickinson and Company, Sparks, MD, USA) for selective enumeration of *Listeria monocytogenes*, and Tryptic Soy Agar supplemented with yeast extract (TSA ± YE) for enumeration of background microflora. For the experiments using PBS as the vehicle, samples were plated onto TSA ± YE. The addition of 0.6% yeast extract to the medium was based on preliminary trials using concentrations of pyruvic acid and/or yeast extract for maximum recovery of injured cells after pressure treatments [11]. Plates were then incubated at 37 °C for 24–48 h. Incubated plates were then manually counted and converted to log values for further descriptive and inferential analyses. The pH values of substrates were measured twice, once after pressure treatment (prior to neutralization) and once before microbiological analyses (after neutralization) using a digital pH meter (Mettler Toledo, AG, Switzerland) calibrated at pH levels of 4.00, 7.01, and 10.01, prior to measurements.

### 2.4. Statistical Analyses and Experimental Design

**Figure 1:**
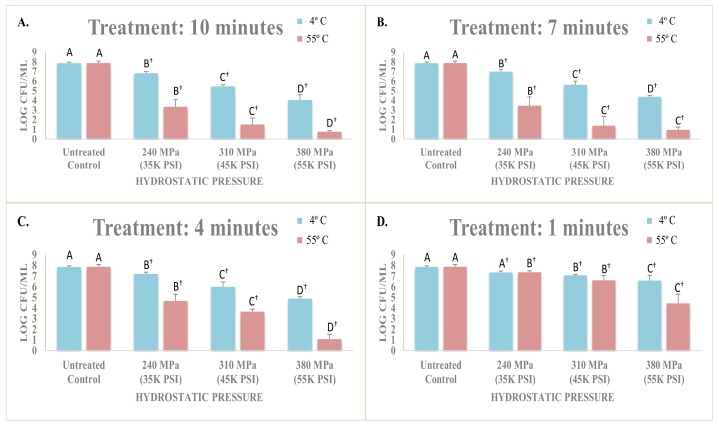
Effects of elevated hydrostatic pressure against four-strain habituated mixture of *Listeria monocytogenes* (ATCC^®^ numbers 51772, 51779, BAA-2657, 13932) in phosphate-buffered saline, treated (Barocycler Hub440, Pressure BioScience Inc., South Easton, MA) at 4 and 55 °C. Within each graph, and for each temperature separately, columns of each pressure intensity level followed by different uppercase letters are representing log CFU/mL values that are statistically (*p <* 0.05) different (Tukey-adjusted ANOVA). Uppercase letters followed by † sign are statistically (*p <* 0.05) different than the untreated control (Dunnett-adjusted ANOVA). (**A**) Treatments for 10 min; (**B**) Treatments for 7 min; (**C**) Treatments for 4 min; (**D**) Treatments for 1 min.

**Figure 2:**
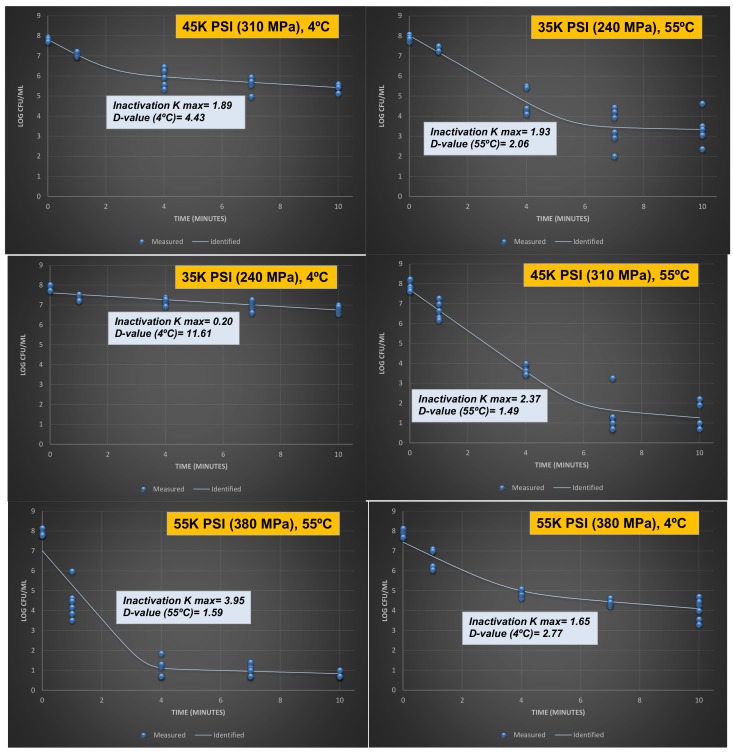
Inactivation rates for four-strain habituated mixture of *Listeria monocytogenes* (ATCC^®^ numbers 51772, 51779, BAA-2657, 13932) exposed to elevated hydrostatic pressure (Barocycler Hub 440, Pressure BioScience Inc., South Easton, MA) in phosphate-buffered saline. K_max_ values are selected from the best-fitted model (goodness-of-fit indicator of R^2^ values, α = 0.05) using the GInaFiT software. K_max_ values are expressions of number of log cycles of reduction in 1/min unit. The *d*-values provided are calculated based on linear model, exhibiting time required for a log (90%) of microbial cell reduction.

**Figure 3:**
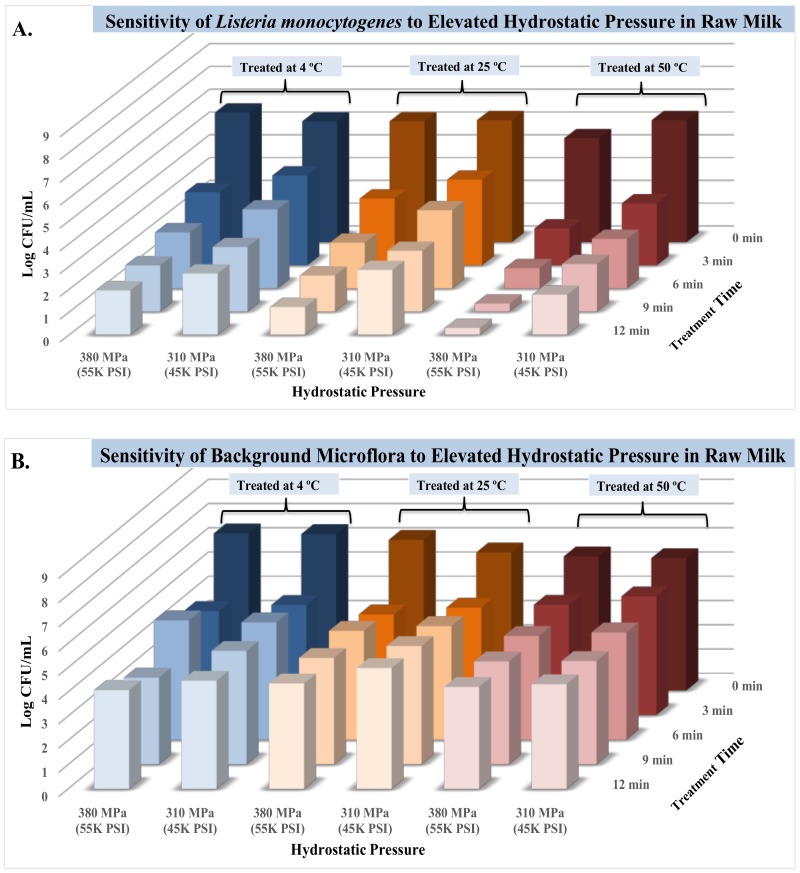
Effects of elevated hydrostatic pressure against four-strain habituated mixtures of *Listeria monocytogenes* (ATCC^®^ numbers 51772, 51779, BAA-2657, 13932) and background microflora in raw milk, treated (Barocycler Hub440, Pressure BioScience Inc., South Easton, MA) at 4, 25, and 50 °C. (**A**) Counts of *Listeria monocytogenes*; (**B**) Counts of background microflora.

**Figure 4:**
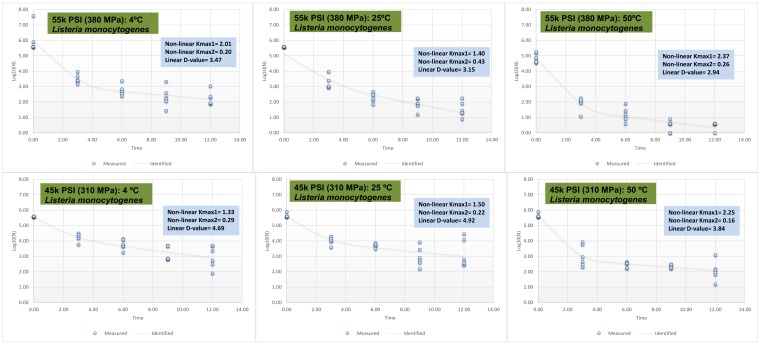
Inactivation indices for four-strain habituated mixture of *Listeria monocytogenes* (ATCC^®^ numbers 51772, 51779, BAA-2657, 13932) exposed to elevated hydrostatic pressure (Barocycler Hub 440, Pressure BioScience Inc., South Easton, MA) in raw milk. K_max_ values are selected from best-fitted biphasic model (goodness-of-fit indicator of R^2^ values, α = 0.05) using the GInaFiT software. K_max_ values are expression of number(s) of log cycles of reduction in 1/min unit, thus larger values correspond to less time needed for microbial cell reductions for each pressure/temperature combination. The *d*-values provided are calculated based on linear model, exhibiting time required for a log (90%) of microbial cell reductions.

Sample sizes of at least 5 repetitions per time/temperature/pressure were obtained based on an a priori power analysis of existing high-pressure pasteurization data of the public health microbiology laboratory using Proc Power of SAS_9.2_ software (SAS Institute, Cary, NC, USA). The current study is a compilation of two separate experiments using PBS (Figure 1 and Figure 2) and raw milk (Figure 3 and Figure 4) as vehicles. These studies were conducted, analyzed, and reported separately. Each experiment was conducted in two biologically independent repetitions, with each repetition considered as a blocking factor in a randomized complete block design. Each block consisted of three replications, and each replication further consisted of two microbiological repetitions. Thus, each reported value is the mean of 12 independent analyses (i.e., 2 blocks, 3 replications, 2 microbiological repetitions per time/pressure/treatment). Data management, log conversion, and descriptive representation of the data were initially conducted in Microsoft Excel. The raw data were then imported to SAS_9.2_ software (SAS Institute, Cary, NC, USA), for inferential statistics at the type I error level of 5% (α = 0.05). For each experiment, an Analysis of Variance (ANOVA) was conducted using the Generalized Linear Model (Proc GLM) of SAS_9.2_ with two mean separation methods. A Tukey adjustment was utilized for pairwise comparisons of all treated samples and controls and further, a Dunnett adjustment was utilized for comparing treated samples with the untreated controls. Microsoft Excel and GInaFiT version 1.7 [15] software (Katholieke Universiteit, Leuven, Belgium) were further used for calculation of inactivation indices (*d*-value and K_max_ values).

## 3. Results and Discussion

### 3.1. Inactivation of Listeria monocytogenes in Phosphate-Buffered Saline

Investigating the sensitivity of the pathogen in phosphate-buffered saline (PBS) medium would provide the opportunity of exploring the synergism of heat and elevated hydrostatic pressure without the interference of intrinsic and extrinsic factors of a food vehicle. These experiments were conducted by inoculating sterilized PBS with the above-mentioned cocktail of the pathogen. The pH of the media prior to inoculation was 7.54 ± 0.1 and were not different (*p* ≥ 0.05) than inoculated samples treated at 4 and 55 °C. Corresponding pH values for inoculated samples at 4 and 55 °C were 7.40 ± 0.1, and 7.44 ± 0.2, respectively. Prior to analyses, samples were kept at refrigeration temperature (4.07 ± 0.2 °C). As further delineated in the Materials and Methods Section 2.2., temperatures before and after treatments were precisely maintained (using a stainless steel jacket connect to refrigerated circulating water bath), monitored (using K-type thermocouples secured inside the chamber wall), and recorded (using HUB PBI 2.3.11 Software). Temperature recordings before and after treatments were similar (*p* ≥ 0.05) and was 3.80 ± 0.2 °C prior to treatments at 4 °C and were recorded as 3.81 ± 0.3 °C at the end of the treatments. The corresponding values for samples’ temperature treated at 55 °C were 54.83 ± 0.4 °C and 55.11 ± 0.4 °C before and after the treatments, respectively. Temperature of the transmission fluid (distilled water), were precisely controlled and monitored at 4 and/or 55 °C as articulated in Section 2.2.

#### 3.1.1. Sensitivity of Listeria monocytogenes in Phosphate-Buffered Saline at 4 °C

Inactivation of *Listeria monocytogenes* counts were investigated at this temperature under three levels of hydrostatic pressure and for time intervals of 0 min (untreated control) up to 10 min. Counts of the pathogen for untreated controls were 7.86 ± 0.1 log CFU/mL (Figure 1). Treatments for one min resulted in no (*p* ≥ 0.05) or only small reductions (*p <* 0.05). As an example, treated samples at 240 MPa, 310 MPa, and 380 MPa after one min had counts of 7.34 ± 0.1, 7.05 ± 0.1, and 6.57 ± 0.1 log CFU/mL (Figure 1D). Longer duration of pressure treatments, predictably resulted in higher inactivation of the pathogen. The counts of *Listeria monocytogenes* were reduced (*p <* 0.05) to 4.88 ± 0.2, 4.38 ± 0.1, and 4.0 ± 0.5 for treatments at 380 MPa after 4, 7, and 10 min, respectively (Figure 1A–C). The corresponding values (*p <* 0.05) at 310 MPa for the above order of treatment times were, 7.05 ± 0.1, 5.99 ± 0.5, and 5.61 ± 0.4, respectively (Figure 1A–C).

#### 3.1.2. Sensitivity of Listeria monocytogenes in Phosphate-Buffered Saline at 55 °C

In exclusion of intrinsic and extrinsic factors of a food vehicle, *Listeria monocytogenes* exhibited great sensitivity to the combination of mild hydrostatic pressure and heat. Even a one-min treatment at 380 MPa and 55 °C was able to reduce (*p <* 0.05) the pathogen counts to 4.44 ± 0.9 log CFU/mL (Figure 1D). With counts of untreated controls at 7.87 ± 0.2 log CFU/mL, this reduction is equivalent to 3.43 log reductions (e.g., >99.9% of the inoculated pathogen). The pathogen counts were further reduced (*p <* 0.05) to <1.07 ± 0.5, <0.95 ± 0.3, and <0.75 ± 0.3 log CFU/mL, for 4-min, 7-min, and 10-min treatments at 380 MPa and 55 °C. Even milder pressure treatments, coupled with elevated heat resulted in an appreciable reduction (*p <* 0.05) of *Listeria monocytogenes*. As an example, 4-min treatments resulted in pathogen counts of 3.66 ± 0.2 log CFU/mL at 310 MPa and 55 °C, and 4.66 ± 0.6 log CFU/mL at 380 MPa and 4 °C. These counts were lower (*p* < 0.05) at 55 °C relative to those samples treated at 4 °C. Pathogen counts for the above two pressures at 55 °C and for 7 min treatments were <1.38 ± 1.0, and 3.45 ± 0.9 log CFU/mL, respectively (Figure 1A–C).

The current challenge study indicates lower levels of pressure coupled with mild heat could be as efficacious as higher levels of pressure at lower temperatures. As an example, counts of *Listeria monocytogenes* were reduced (*p <* 0.05) from 7.86 ± 0.1 to 4.88 ± 0.2 after a 380 MPa treatment at 4 °C. Similarly, *Listeria monocytogenes* counts were reduced (*p <* 0.05) from 7.87 ± 0.2 to 4.66 ± 0.6 after a 240 MPa treatment at 55 °C (Figure 1A–C). Treatments of 3 to 5 min are current standard procedures in the manufacturing of food products using pressure-based technologies [10].

#### 3.1.3. Inactivation Indices of Listeria monocytogenes in Phosphate-Buffered Saline

Calculation of inactivation indices not only improves the adaptability of a challenge study by private industry but further delineates the synergism of heat and hydrostatic pressure for decontamination of *Listeria monocytogenes* (Figure 2). The index *d*-value is obtained based on a linear model, corresponding to the time (in this study in a unit of min) required at specific conditions (pressure, heat, and other intrinsic, and extrinsic factors) to reduce 90% of the exposed microorganism [16]. Under the condition of this experiment, we observed a *d*-value of 2.77 min for inactivation of *Listeria monocytogenes* at 4 °C and at 380 MPa. The value at the same level of pressure but at 55 °C was reduced to 1.59 min (Figure 2). Similar synergism was observed at lower pressures. The *d*-values were 4.43 and 1.49 for treatments at 310 MPa at 4 and 55 °C, respectively and were 11.61 and 2.06 min for treatments at 240 MPa at 4 and 55 °C, respectively. This indicates that a treatment at lower pressure and higher temperature (310 MPa at 55 °C), could be comparable (*p* ≥ 0.05) to a treatment at higher pressure and lower temperature (380 MPa at 4 °C). This synergism could be of great importance to manufacturing facilities using the technology to lower the cost of operation since lower levels of pressure had been associated with lower maintenance cost and higher shelf-life of the pressure vessels [17]. The cost associated with high-pressure processing is currently the main barrier for further adoption of this technology in the private industry [10].

Recent studies also delineate that alternative inactivation indices, particularly those obtained based on non-linear models, could be of great importance for stakeholders since the microbial reduction of many food-pathogen combinations may not follow a linear pattern [11]. As further elaborated in the Materials and Methods Section 2.4., the current study is reporting the non-linear inactivation index K_max_ calculated based on the best-fitted (maximum R^2^) model. In contrast to the *d*-value, this index has a unit of 1/min, thus, larger K_max_ values are corresponding to higher/faster microbial inactivation [18]. The K_max_ values at 4 °C were 1.65, 1.89, and 0.20 for treatments at 380 MPa, 310 MPa, and 240 MPa, respectively. The values were increased to 3.95, 2.37, and 1.93 for the above order of the pressure treatments when tested at 55 °C (Figure 2).

### 3.2. Inactivation of Listeria monocytogenes and Natural Microflora in Raw Milk

Relative to challenge studies conducted in phosphate-buffered saline medium, studying the inactivation of *Listeria monocytogenes* in raw milk could provide a more realistic interpretation, particularly when such studies conducted in presence of natural microflora of the product with the existence of intrinsic and extrinsic factors that had not been altered by any previous treatment. 

#### 3.2.1. Sensitivity of Listeria monocytogenes and Natural Microflora in Raw Milk at 4 °C

Under the condition of this experiment, the pH of raw milk samples treated at the various time and pressure intensity levels were similar (*p* ≥ 0.05) and ranged from 6.72 ± 0.1 to 6.85 ± 0.1. The pH of untreated raw milk was 6.82 ± 0.1, while the pH of treated milk neutralized in D/E broth prior to microbiological analyses was 7.23 ± 0.1. The temperature recordings of the treatments remained constant (*p* ≥ 0.05) before and after treatments, ranging from 3.58 ± 0.3 °C to 3.93 ± 0.3 °C and 3.70 ± 0.4 °C to 3.95 ± 0.1 °C for before and after the pressure treatments, respectively. Counts of selective medium (PALCAM agar), associated with *Listeria monocytogenes* showed the standard deviation of 0.1 to 0.9 (average standard deviation of 0.3) and are summarized in Figure 3A. The pathogen count of untreated controls at 4 °C was 5.30 ± 0.1 log CFU/mL, the count was reduced by 3.35 log for treatment at 380 MPa for 12 min to 1.95 ± 0.4 log CFU/mL. The corresponding log reductions for 9-, 6- and 3-min treatments at 380 MPa were 3.3, 2.8, and 2.1, respectively, exhibiting in excess of a 99% reduction of the pathogen at 380 MPa treatments. At lower pressure intensities of 310 MPa, these reductions showed similar trends: Treatments for 12, 9, 6, and 3 min at this intensity level lead to 2.6, 2.5, 1.8, and 1.4 log reductions (*p <* 0.05), respectively. 

Figure 3B, summarizes counts obtained from non-selective media (TSA supplemented with yeast extract) corresponding to inactivation of background microflora. These counts exhibited standard deviations ranging from 0.1 to 0.8 (average standard deviation of 0.3). In the vast majority of the time-pressure combinations at 4 °C, background microflora counts of the raw milk were less sensitive to treatments relative to the inoculated pathogen. As an example, treatments for 12 min, modestly reduced (*p* < 0.05) the background microflora for 2.03 and 1.99 log, for treatment intensity of 380 MPa and 310 MPa, respectively. The higher tolerance of background microflora to elevated hydrostatic pressure had been previously reported in similar products and could be primarily attributed to the presence of spore-forming organisms that are ubiquitous in production and manufacturing environments [19].

#### 3.2.2. Sensitivity of Listeria monocytogenes and Natural Microflora in Raw Milk at 25 °C

Similar to the samples treated at 4 °C, samples treated at 25 °C had comparable (*p* ≥ 0.05) pH counts ranging from 6.67 ± 0.1 to 6.77 ± 0.1. Temperature recordings prior and after treatments were controlled at 25 °C (*p* ≥ 0.05) and were ranging from 24.10 ± 0.8 to 24.67 ± 0.7 and 23.83 ± 0.5 to 24.63 ± 0.9, prior and after the pressure treatments, respectively. Pathogen load (PALCAM counts) were 5.30 ± 0.1 log CFU/mL (Figure 3A) and were reduced (*p <* 0.05) to 1.58 ± 0.1 log CFU/mL after pressure treatment of 380 MPa for 12 min. Similarly, 3.29 and 2.27 log reductions (*p <* 0.05) were observed for treatments of 9 and 6 min at 380 MPa, respectively (Figure 3A). More than 99% of the inoculated *Listeria monocytogenes* was inactivated at lower pressure of 310 MPa, as result of 12- and 9-min treatments (Figure 3B). Background microflora counts at this temperature were more fastidious to the pressure treatments relative to pathogen counts (Figure 3B). As an example, only 1.86 and 0.81 log reductions were achieved after 12 min of treatment at 380 MPa, and 310 MPa, respectively. Minor differences were observed during inactivation of the pathogen and background microflora comparing treatments of 4 and 25 °C, indicating that these temperatures might be used interchangeably in manufacturing facilities and during validation studies. Reductions obtained in this study are in harmony with previous studies at similar temperatures and pressure intensity levels, where 2.11 log reductions of *Listeria monocytogenes* were reported at 500 MPa after 10 min of treatment of milk at 20 °C [20]. Similar trends were also reported for inactivation of *Listeria monocytogenes* at 400 MPa at temperatures of 20 to 25 °C [21,22]. Studying inactivation of *Listeria monocytogenes* at lower and high temperatures combined with mild elevated hydrostatic pressure, is currently a knowledge gap of hurdle validation data against this pathogen as well as those conducted against the natural background flora of raw milk. A pressure treatment coupled with mild temperature of 50 or 55 °C, could assure microbiological safety of a product while reducing cost associated with pressure vessels shelf-life and high pressure pasteurization maintenance [10].

#### 3.2.3. Sensitivity of Listeria monocytogenes and Natural Microflora in Raw Milk at 50 °C

Synergism of temperature at 50 °C with elevated hydrostatic pressure resulted in most appreciable reductions (*p <* 0.05) in counts of *Listeria of monocytogenes*. Selective counts, corresponding with the inoculated pathogen (PALCAM counts) were 4.56 ± 0.3 log CFU/mL prior to treatment at 380 MPa (Figure 3A). The counts were reduced (*p <* 0.05) to below the detection limit after 12 min of treatment at 380 MPa/ 50 °C. Similarly, 4.21, 3.67, and 2.08 log reductions (*p <* 0.05) were observed for 9-, 6-, and 3-min treatments at this pressure and temperature combination, respectively (Figure 3A). Treatments of 310 MPa similarly resulted in log reductions (*p <* 0.05) ranging from 2.75 to 3.59 (Figure 3A). Unlike the inoculated pathogen, the natural microflora was affected modestly (*p <* 0.05) even at 50 °C, for 12-min treatments at 380 MPa, exhibiting 1.32 log reductions (Figure 3B). The corresponding log reductions associated with 9-, 6-, and 3-min treatments at 380 MPa/ 50 °C were 1.28, 1.25, and 0.98, respectively, for the natural microflora (Figure 3B). These reductions were also modest at lower pressure intensities, as an example, 1.13 log reductions (*p <* 0.05) were observed as result of pressure treatments at 310 MPa at 50 °C, after 12 min (Figure 3B). The resistance of spoilage microorganisms and natural microflora of the raw milk could be attributed to the presence of spore-forming organisms that are ubiquitous in processing area [11] as previously delineated in Section 3.2.1.

### 3.3. Synergism of Elevated Hydrostatic Pressure and Heat for Inactivation of Listeria monocytogenes and Natural Microflora

Current data exhibit strong synergism between mild heat and hydrostatic pressure for inactivation of *Listeria monocytogenes* in raw milk. The synergism among extrinsic factors of food against microbial pathogen had been previously discussed as part of “hurdle technology” [23]. With the assumption of a linear relationship between reduction of *Listeria monocytogenes* in raw milk as affected by elevated hydrostatic pressure and mild heat, our study shows 3.47 min are required (Figure 4) at pressure levels of 380 MPa at 4 °C for a 90% reduction of the pathogen (i.e., *d*-value = 3.47). These corresponding *d*-values were reduced to 3.15 and 2.94 at 25 and 50 °C (Figure 4). The assumption of non-linearity and using a best-fitted model obtained by GInaFiT software [15] exhibited similar trends (Figure 4). One of the main challenges for current manufacturers of pressure-treated products is slightly higher production costs relative to conventional products treated solely by thermal processing [10,11]. The current study indicates that pressure treatments at lower intensities, such as 380 MPa and 310 MPa alone or coupled with mild heat could be an alternative to pressures at very high levels of hydrostatic pressure. The lower pressure treatments are typically associated with increased shelf-life of the pressure vessels and reduced cost of pressure processing [17]. Similar synergism was observed for inactivation of natural microflora, although the extent of reductions were less than inactivation rates observed for the pathogen (Figure 3B). As previously discussed, this is due to presence of spore-forming organisms that are ubiquitous in raw milk production and manufacturing area and are considered to be resistant to pressure-based treatments [11].

## 4. Conclusions

Under the conditions of our experiments, we observed *d*-values of 2.77, 4.43, and 11.61 for inactivation of *Listeria monocytogenes* treated at 380 MPa, 310 MPa, and 240 MPa treated in PBS at 4 °C. Corresponding values for pressure treatments in PBS at 55 °C were appreciably lower, delineating strong synergism between heat and hydrostatic pressure for inactivation of *Listeria monocytogenes*. Similar synergistic effects were demonstrated against *Listeria monocytogenes* inoculated in raw milk. Reducing the cost associated with pressure-based technologies are currently the main challenge for further adoption of this emerging technology in various sectors of food manufacturing. The current study delineated the pressure treatments at lower intensity levels, coupled with mild heat could result in an appreciable reduction of *Listeria monocytogenes*. This synergism was demonstrated in laboratory media with the exclusion of intrinsic and extrinsic factors of a food vehicle as well as in studies conducted in raw milk. The synergistic effect of mild heat and pressure-based treatments could be of assistance to manufacturers for avoiding extreme pressures of 600 MPa or above that are typically associated with reduced shelf-life of the pressure vessels, increased manufacturing costs, and increased concern for reduction of the nutritive value of the food.
